# Altered Lung Morphogenesis, Epithelial Cell Differentiation and Mechanics in Mice Deficient in the Wnt/β-Catenin Antagonist Chibby

**DOI:** 10.1371/journal.pone.0013600

**Published:** 2010-10-25

**Authors:** Damon Love, Feng-Qian Li, Michael C. Burke, Benjamin Cyge, Masao Ohmitsu, Jeffrey Cabello, Janet E. Larson, Steven L. Brody, J. Craig Cohen, Ken-Ichi Takemaru

**Affiliations:** 1 Department of Pharmacological Sciences, SUNY at Stony Brook, Stony Brook, New York, United States of America; 2 Graduate Program in Molecular and Cellular Pharmacology, SUNY at Stony Brook, Stony Brook, New York, United States of America; 3 Medical Scientist Program (MSTP), SUNY at Stony Brook, Stony Brook, New York, United States of America; 4 Graduate Program in Genetics, SUNY at Stony Brook, Stony Brook, New York, United States of America; 5 Section of Neonatology, Department of Pediatrics, SUNY at Stony Brook, Stony Brook, New York, United States of America; 6 Division of Pulmonary and Critical Care, Department of Internal Medicine, Washington University School of Medicine, St. Louis, Missouri, United States of America; Cincinnati Children's Hospital Medical Center, United States of America

## Abstract

The canonical Wnt/β-catenin pathway plays crucial roles in various aspects of lung morphogenesis and regeneration/repair. Here, we examined the lung phenotype and function in mice lacking the Wnt/β-catenin antagonist Chibby (Cby). In support of its inhibitory role in canonical Wnt signaling, expression of β-catenin target genes is elevated in the *Cby^−/−^* lung. Notably, Cby protein is prominently associated with the centrosome/basal body microtubule structures in embryonic lung epithelial progenitor cells, and later enriches as discrete foci at the base of motile cilia in airway ciliated cells. At birth, *Cby^−/−^* lungs are grossly normal but spontaneously develop alveolar airspace enlargement with reduced proliferation and abnormal differentiation of lung epithelial cells, resulting in altered pulmonary function. Consistent with the Cby expression pattern, airway ciliated cells exhibit a marked paucity of motile cilia with apparent failure of basal body docking. Moreover, we demonstrate that Cby is a direct downstream target for the master ciliogenesis transcription factor Foxj1. Collectively, our results demonstrate that Cby facilitates proper postnatal lung development and function.

## Introduction

The morphogenesis of lungs is dependent upon intricate interactions between the endodermally derived respiratory epithelium and the surrounding mesenchyme, and involves a complex network of signal transduction events initiated by several families of secreted factors [1], [2]. One such signaling pathway, the canonical Wnt/β-catenin pathway, has been shown to play a crucial role in normal lung development and homeostasis [1], [3], [4].

Intracellular signaling activated by the Wnt family of secreted cystein-rich glycoproteins is pivotal for embryonic development, stem cell self-renewal and adult homeostasis [5], [6]. Perturbations in Wnt signaling have been linked to a wide range of human diseases [7], [8], [9]. The best understood canonical Wnt pathway utilizes nuclear β-catenin as a transcriptional coactivator that stimulates gene expression by binding to the T-cell factor/lymphoid enhancer factor (Tcf/Lef) family of transcription factors [10], [11]. Multiple Wnt ligands and Frizzled receptors are differentially expressed in the developing and adult lung, and gain- and loss-of-function studies in mice confirm the importance of Wnt signaling in regulating diverse aspects of lung morphogenesis [4], [12]. Wnt/β-catenin signaling has been conditionally inactivated in embryonic lung epithelial cells in mice, resulting in enhanced specification of proximal lung and a failure of formation of distal lung structures [13], [14]. On the other hand, sustained activation of β-catenin signaling specifically in the developing lung disrupts epithelial cell differentiation, causing enlargement of peripheral air spaces [15], [16]. More recently, the Wnt/β-catenin pathway has been shown to control lung stem cell expansion and regeneration/repair [17], [18]. Given the essential role of Wnt signaling in the development and maintenance of the tissue, it is not surprising that this pathway has been associated with various lung diseases including lung cancer and pulmonary fibrosis [4], [7], [12].

Chibby (Cby) is a 15-kDa protein evolutionarily conserved from fly to human [19]. We demonstrated that Cby physically interacts with β-catenin to repress β-catenin-dependent gene activation [19], [20], [21], [22]. The majority of *Cby^−/−^* mice die in the early postnatal period [23]. Throughout life, surviving *Cby^−/−^* mice suffer from chronic upper respiratory tract infection caused by a complete absence of mucociliary transport activity. Our studies further revealed the presence of poorly differentiated ciliated cells characterized by a marked decrease in the number of motile cilia on nasal epithelial cells of *Cby^−/−^* mice, although the ultrastructure of the axonemes appears normal. In accordance with these findings, Cby protein localizes to the ciliary base of motile cilia in the nasal epithelium [23], suggesting that Cby is directly involved in motile ciliogenesis. The phenotypes of *Cby^−/−^* mice share similarities to clinical features of primary ciliary dyskinesia (PCD) [24], [25].

In the present study, we describe the characterization of lung morphology and mechanics in *Cby^−/−^* mice. Consistent with Cby being a Wnt/β-catenin antagonist, β-catenin signaling is moderately elevated in *Cby^−/−^* lungs. Upon birth, *Cby^−/−^* lungs appear histologically indistinguishable from those of *Cby^+/+^* littermates but progressively develop alterations in lung architecture and differentiation marker expression. During early lung development, intense Cby localization is predominantly detected at the centrosome and basal body of primary cilia in epithelial progenitor cells, and later at the ciliary base in airway ciliated cells. In good agreement with this, *Cby^−/−^* mice display a low abundance of motile cilia in large airways. Furthermore, we show that Cby expression is directly up-regulated by Foxj1. Our findings therefore suggest that the *Cby* gene is essential for proper lung development and function during postnatal life.

## Results

### Ablation of Cby results in elevation of Wnt/β-catenin signaling in the lung

**Figure 1:**
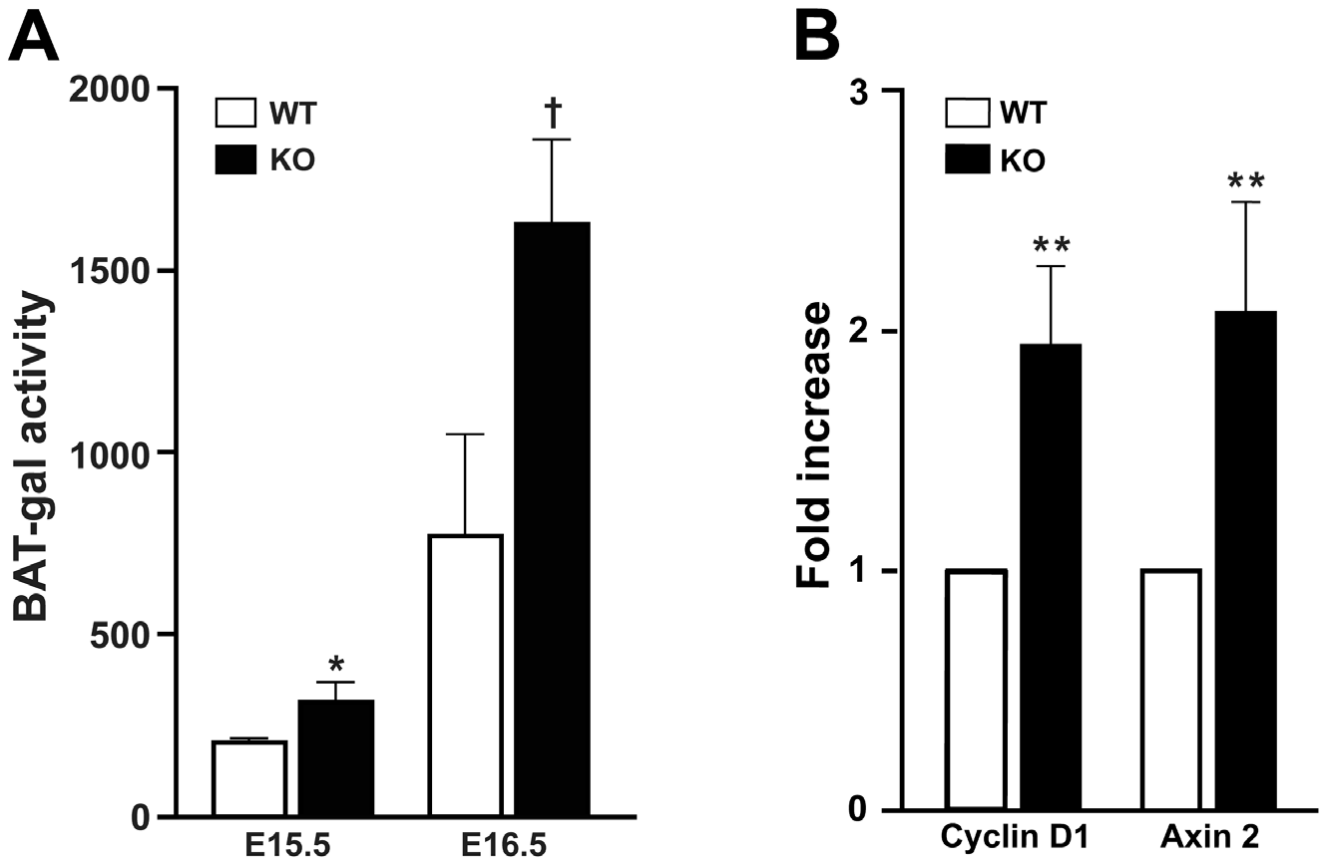
Wnt/β-catenin signaling activity is elevated in *Cby^−/−^* lungs. (**A**) BAT-gal reporter activity was measured in lung homogenates from *Cby^+/+^*and *Cby^−/−^* embryos carrying the BAT-gal transgene at E15.5 and E16.5 (n = 3 per genotype per embryonic stage), and normalized to total protein concentration as determined by the Bradford assay. Values are expressed as mean β-galactosidase activity units per microgram of protein ± SE. Student's *t*-test; *P<0.05, †*P*<0.01. (**B**) Real-time PCR analysis was performed for expression levels of the direct β-catenin target genes *cyclin D1* and *axin2* in adult *Cby^+/+^*and *Cby^−/−^* lungs (n = 3 per genotype). WT values were set as 1. Values represent means ± SE. Student's *t*-test, **P<0.001.

We previously demonstrated that Cby physically interacts with β-catenin to inhibit β-catenin-dependent transcriptional activation [19], [21], [22]. In fact, depletion of Cby leads to ectopic activation of β-catenin signaling in multiple experimental systems [19], [23]. Given the critical role of the Wnt/β-catenin pathway for lung development, we first examined whether Wnt/β-catenin signaling is affected in *Cby^−/−^* lungs. To this end, we employed BAT-gal reporter mice that express the *lacZ* gene under the control of β-catenin-responsive elements [26]. *Cby^+/+^* and *Cby^−/−^* embryos carrying the BAT-gal transgene were harvested at embryonic day (E) 15.5 and E16.5, and lung lysates prepared for the measurement of β-galactosidase activity. As shown in Figure 1A, BAT-gal activity in *Cby^+/+^* lungs was relatively low at E15.5 but increased towards E16.5. In *Cby^−/−^* lungs, elevated levels of BAT-gal activity were seen compared to *Cby^+/+^* controls at both E15.5 (*P*<0.05) and E16.5 (*P*<0.01), consistent with Cby being a negative regulator of the Wnt/β-catenin pathway. In order to independently confirm these results, we evaluated expression levels of the direct β-catenin target genes *cyclin D1* and *axin2* in adult lungs using real-time PCR. There was a mild but consistent increase (about 2-fold) in the expression of these genes in *Cby^−/−^* lungs in comparison with *Cby^+/+^* controls (Figure 1B). These data indicate that Cby negatively regulates Wnt/β-catenin signaling in the lung.

### Cby expression during lung development

**Figure 2:**
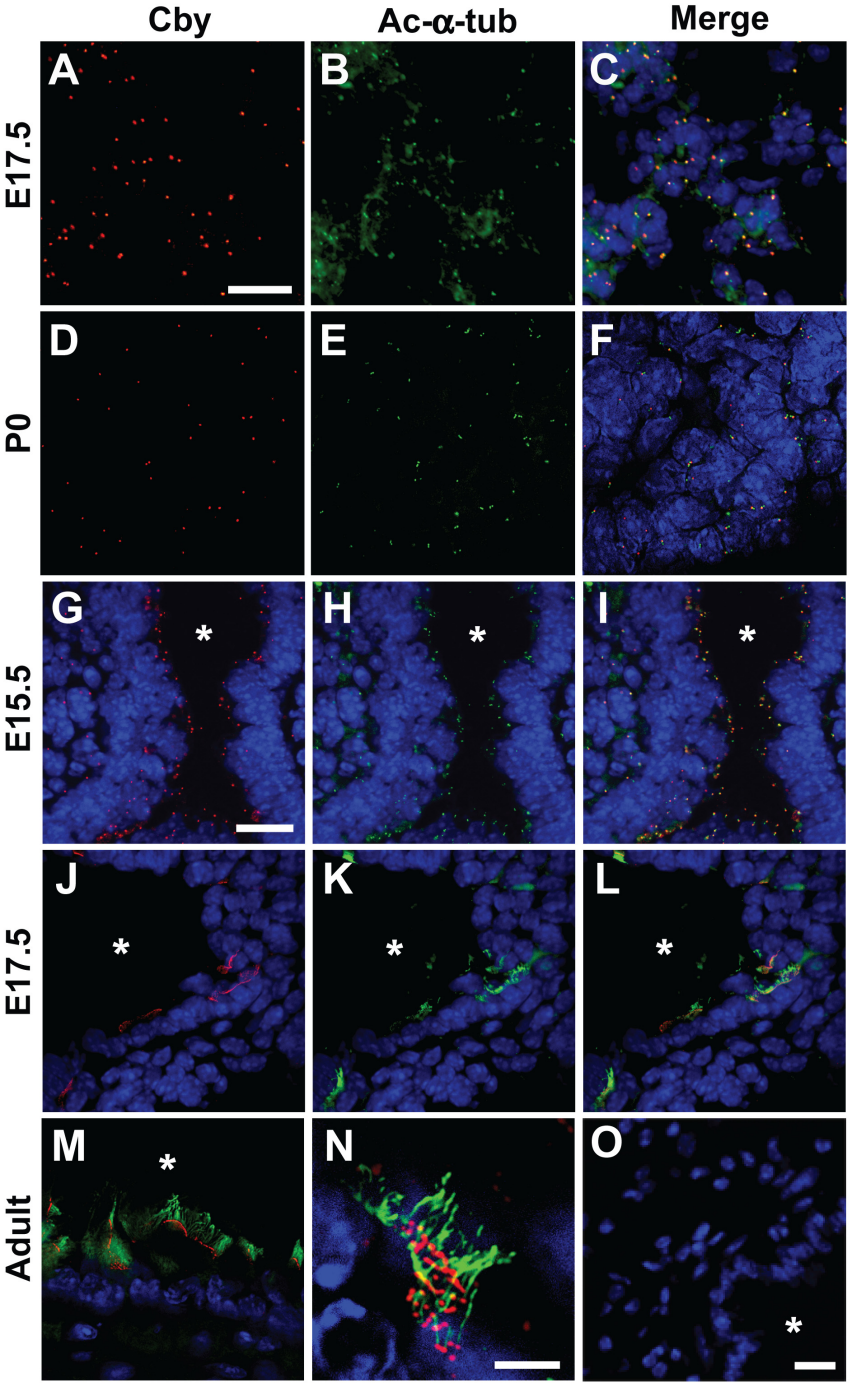
Cby protein localization in the lung. (**A–F**) Peripheral lung sections from E17.5 and P0 *Cby^+/+^* lungs were co-immunostained for Cby (red) (**A, D**) and the centrosomal/ciliary maker acetylated-α-tubulin (green) (**B, E**), and the merged images are shown in (**C, F**). (**G–N**) Lung airway sections from E15.5, E17.5 and adult *Cby^+/+^* lungs were double-labeled with antibodies against Cby (red) (**G, J**) and acetylated-α-tubulin (green) (**H, K**), and the merged images are shown in (**I, L, M, N**). High-magnification view shows that Cby protein appears as discrete foci at the base of motile cilia (**N**). Nuclei were stained with DAPI. (**O**) Lung sections from adult *Cby^−/−^* mice were stained with the anti-Cby antibody, followed by Alexa Fluor 568-conjugated secondary antibody (red) as a specificity control. Asterisks indicate the airway lumen. Scale bars: (**A–F**) 10 µm; (**G–M**) 10 µm; (**N**) 2 µm; (**O**) 2 µm.

Through RT-PCR and western blot analysis, we found that Cby is expressed in embryonic (E14.5 and E17.5) and postnatal (postnatal day (P) 7, P21 and adult) lungs (Figure S1). To gain insights into the localization of Cby protein in the lung, we conducted immunofluorescent staining using anti-Cby antibody [23]. In the developing peripheral lung at E17.5 (Figure 2A–C) and P0 (Figure 2D–F), Cby protein predominantly localized to punctate perinuclear foci positive for the centrosomal/ciliary marker acetylated-α-tubulin, which most likely represented centrosomes. Some of the Cby-positive cells appear to be immature type II cells (Figure S2A). These discrete foci intensely labeled with Cby or acetylated-α-tubulin were no longer noticeable in adult peripheral lungs (data not shown). In the developing large airway epithelium at E15.5 (Figure 2G–I), Cby was detected as punctate signals within the cytoplasm that partially colocalized with acetylated-α-tubulin, clustering along the airway lumen. Some of these Cby-positive organelles could be basal bodies of transient primary cilia in immature airway epithelial cells as reported recently [27]. In fact, Foxj1-expressing ciliated cell precursors contained discrete dots of Cby staining in the apical region (Figure S2B). At E17.5, Cby protein was detectable intensely at motile cilia visualized with anti-acetylated-α-tubulin antibody in differentiating ciliated cells (Figure 2J–L). The ciliary localization of Cby persisted in fully differentiated ciliated cells in adult airways (Figure 2M), and close examination indicated that Cby positions at the base of motile cilia (Figure 2N). This is consistent with our prior findings that Cby localizes to the ciliary base at the apical surface of nasal ciliated cells [23]. Taken together, our data suggest that Cby protein is highly concentrated in centrosome and basal body microtubule structures in different cell types throughout lung development.

### *Cby^−/−^* mice exhibit alveolarization defects

**Figure 3:**
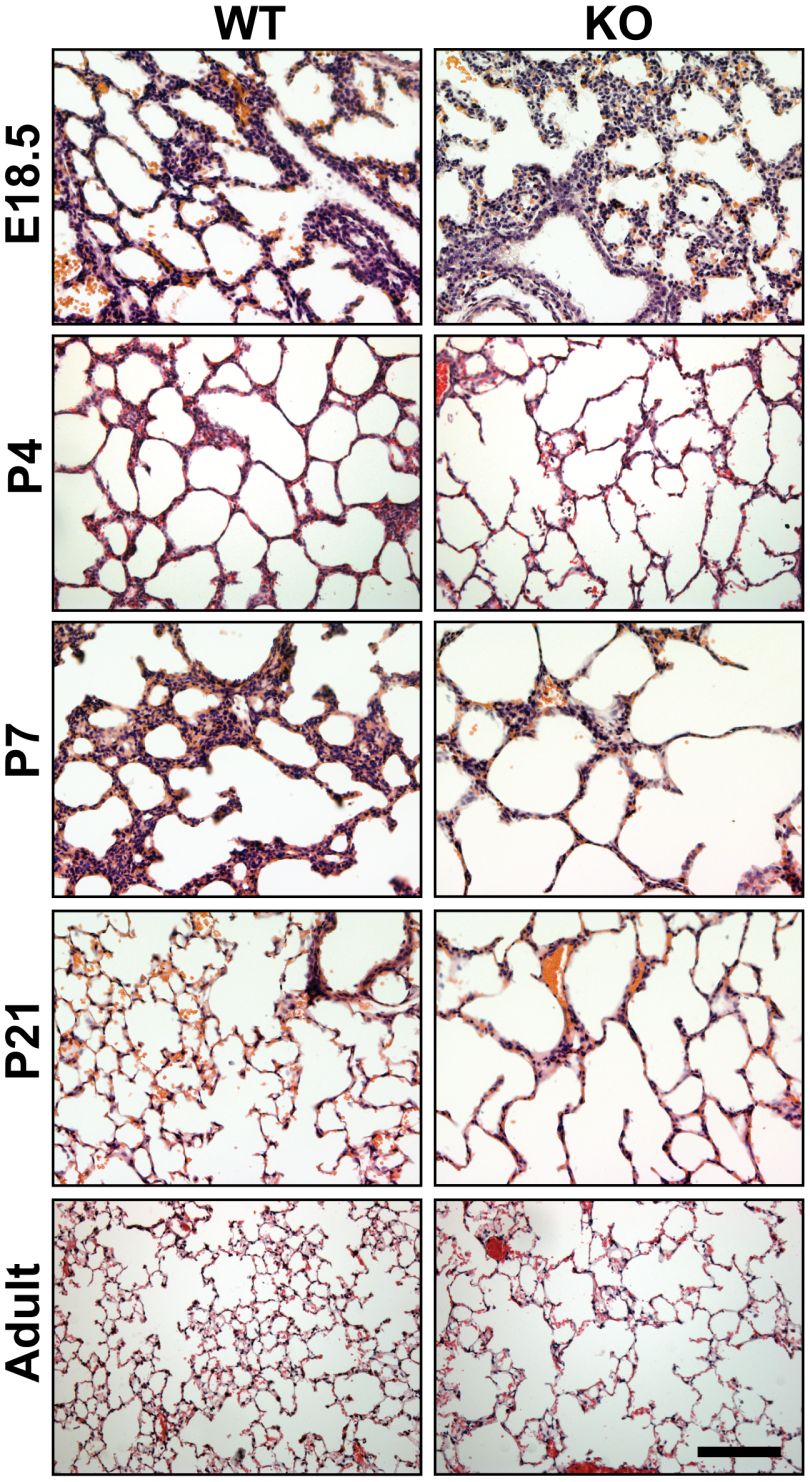
Alveolar airspace enlargement in *Cby^−/−^* lungs. Peripheral lung sections were obtained from *Cby^+/+^* and *Cby^−/−^* mice at the indicated ages, and stained with hematoxylin and eosin (H&E). Images presented here are representative of at least 3 animals per genotype per age. Scale bar, 200 µm.

On the C57BL/6 genetic background used throughout this study, *Cby^−/−^* neonates seem largely normal at birth but fail to gain weight, and about 80% show early postnatal death before or shortly after weaning [23]. The surviving *Cby^−/−^* animals grow into adults and appear grossly normal with a slightly reduced body size. We did not notice significant differences in the lung-to-body weight ratios between *Cby^−/−^* and *Cby^+/+^* control littermates at all time points examined. Upon histological examination at E18.5 and P4, lung morphology was not significantly perturbed in *Cby^−/−^* mice compared to *Cby^+/+^* controls (Figure 3). In contrast, as alveologenesis continued postnatally, enlarged distal airspaces were evident in *Cby^−/−^* lungs as early as P7, and persisted in adult lungs. Despite the marked changes in lung architecture, there were no obvious signs of inflammation, infection or fibrosis in the lungs of *Cby^−/−^* mice up to 18 months of age (data not shown).

**Figure 4:**
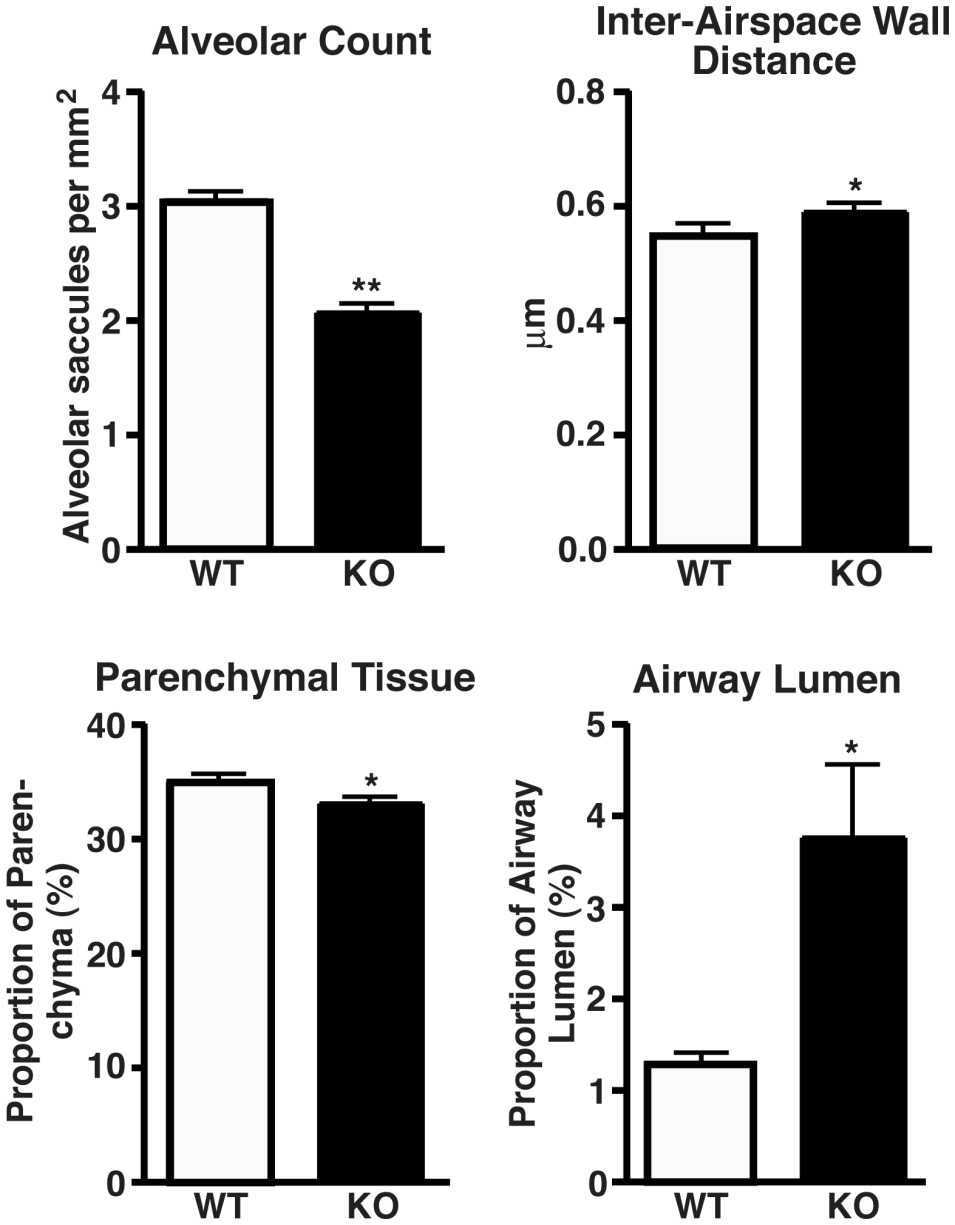
Morphometric analysis of air-exchanging parameters. The alveolar saccules per mm^2^, inter-airspace wall distance (Lm), proportion of the lung composed of parenchymal tissue, and airway luminal area relative to the total lung area were analyzed in the lungs from 10- to 13-week-old *Cby^−/−^* (*n* = 4) and *Cby^+/+^* (*n* = 4) animals by two investigators blinded to genotype. Values are means ± SE. Student's *t*-test; *P<0.05, **P<0.001.

To precisely quantify changes in lung structure, we performed morphometric studies on the lungs from *Cby^−/−^* and *Cby^+/+^* adult animals. The complexity of the lung parenchymal tissue was measured by comparing the following three parameters: the number of alveolar saccules per mm^2^; the relative amount of parenchymal tissue; and the inter-airspace wall distance [mean linear intercept (Lm)], which measures the distance between alveolar walls. In agreement with our histological findings, morphometric analysis revealed a 33% decrease in the number of alveolar saccules per mm^2^ in *Cby^−/−^* lungs compared to *Cby^+/+^* (2.05±0.077 vs. 3.04±0.082; *P*<0.001) (Figure 4). The gas-exchange surface area was reduced in *Cby^−/−^* lungs in comparison with *Cby^+/+^* control lungs as observed by the increased mean distance between alveolar walls (0.59±0.014 µm vs. 0.55±0.016 µm; *P*<0.05) as well as by the reduced percentage of lung parenchymal tissue (33.0±0.57% vs. 34.9±0.61%; *P*<0.05). Notably, the proportion of lung airway lumen to the total lung area was significantly greater in *Cby^−/−^* mice than that in *Cby^+/+^* mice (3.76±0.79% vs. 1.24±0.14%; *P*<0.05). Therefore, our comprehensive morphometric studies conclude that loss of Cby results in airspace enlargement and increased airway luminal area.

### Decreased cell proliferation in postnatal *Cby^−/−^* lungs

**Figure 5:**
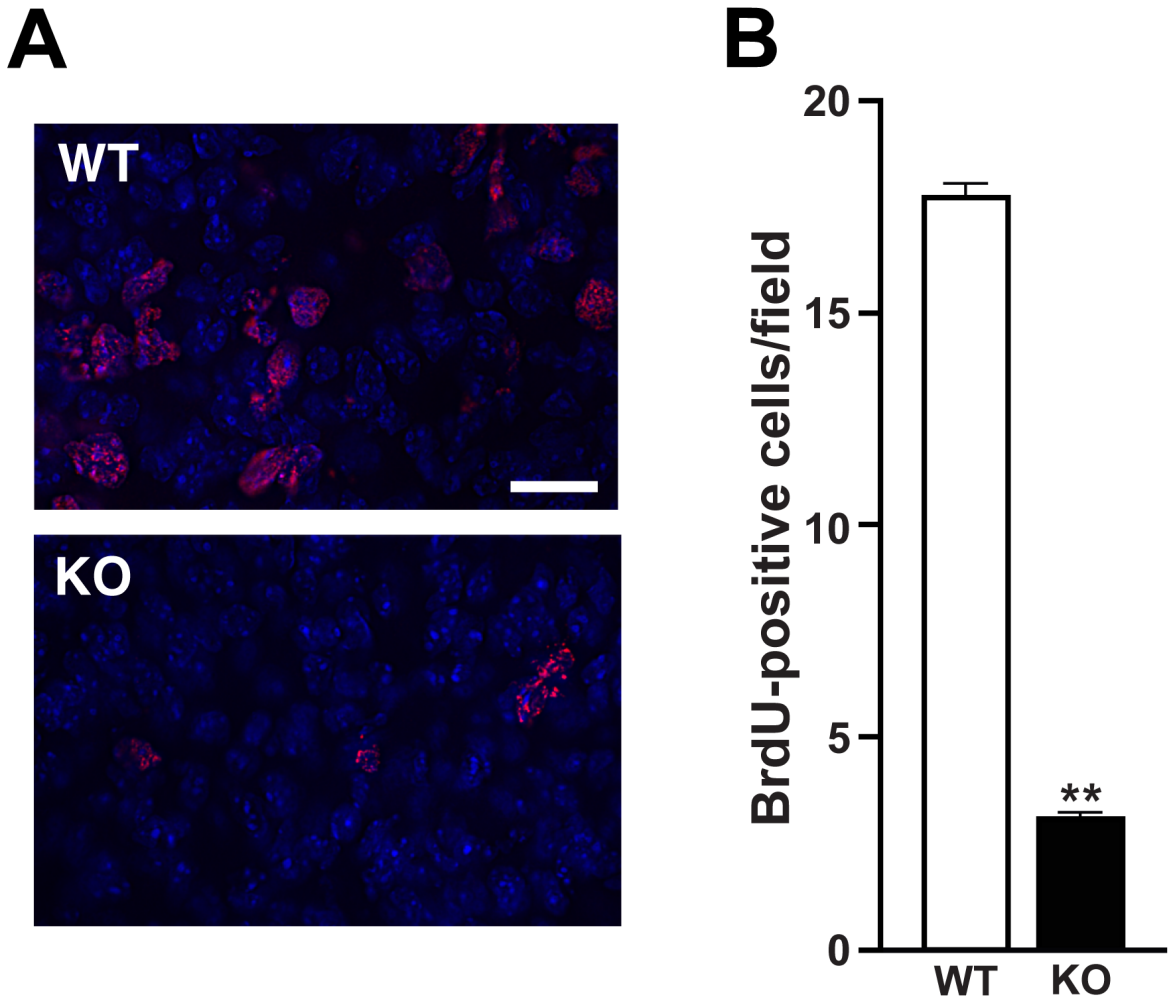
Reduced cell proliferation in postnatal *Cby^−/−^* lungs. (**A**) Representative immunofluorescent images of BrdU incorporation in P11 lungs are shown. Nuclei were visualized with DAPI. Scale bar, 2 µm. (**B**) Quantification of BrdU-positive cells shows a dramatic reduction in cell proliferation in *Cby^−/−^* lungs at P11 (n = 3 per genotype). Values are means ± SE. Student's *t*-test; **P<0.001.

To gain insights into the hypoplastic lung phenotype of *Cby^−/−^* mice, we assessed cell proliferation and apoptosis in the postnatal lung at P11 when alveolarization is actively taking place. BrdU incorporation assays revealed a dramatically reduced number of proliferating cells in *Cby^−/−^* lungs (Figure 5). No significant difference in apoptosis was detected by immunostaining for activated caspase-3 (data not shown). It has been demonstrated that α-smooth muscle actin-positive myofibroblasts are located at the tips of newly forming, immature alveolar septa, and play important roles in septal formation [28]. However, immunofluorescent staining for α-smooth muscle actin showed a normal staining pattern in *Cby^−/−^* lungs at P11 (data not shown). These results suggest that the lung phenotype of *Cby^−/−^* mice is attributable, at least in part, to reduced proliferation rather than increased apoptosis or alveolar septation defects.

### Aberrant differentiation of alveolar epithelial cells in *Cby^−/−^* mice

**Figure 6:**
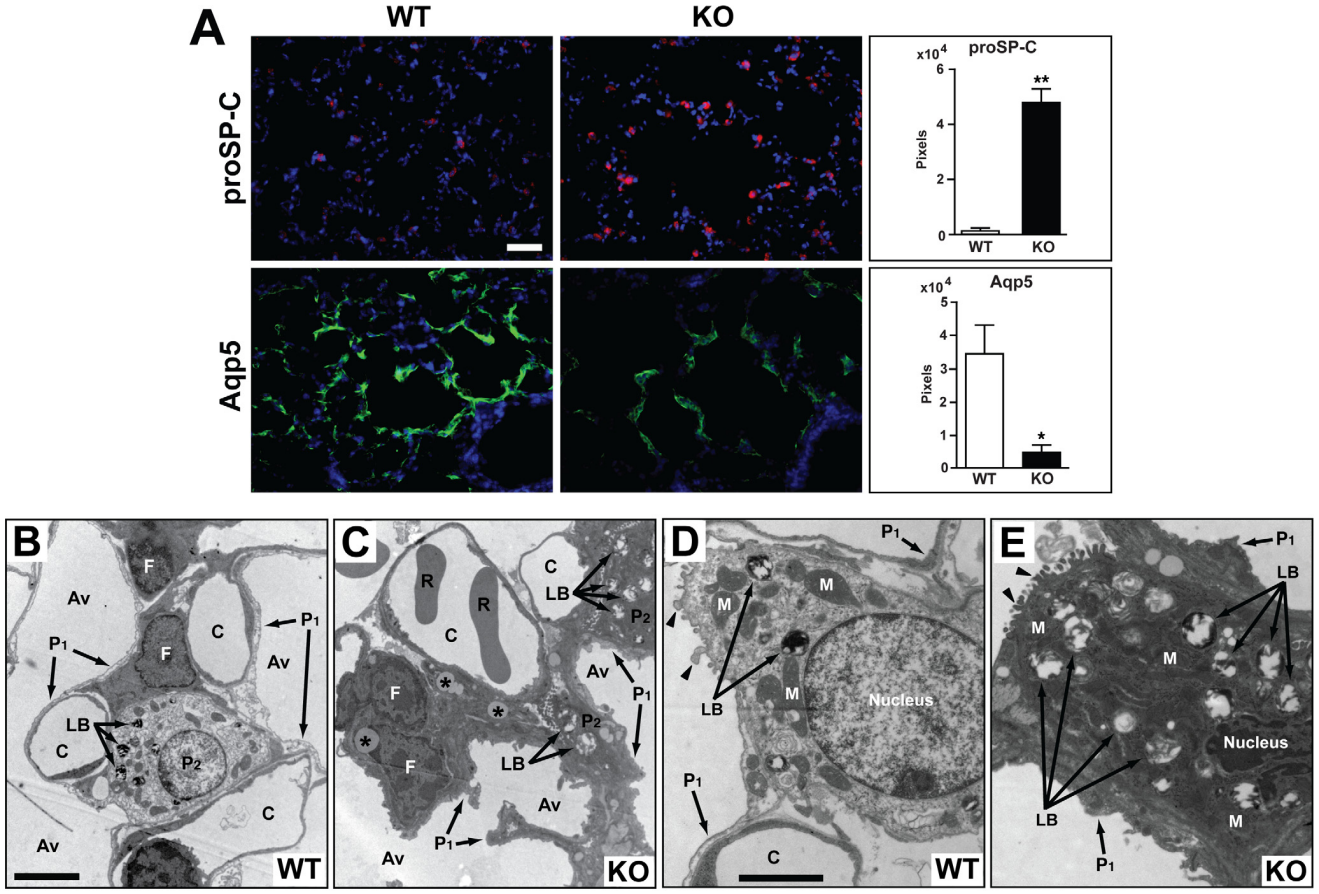
Defective differentiation and morphology of alveolar epithelial cells in *Cby^−/−^* mice. (**A**) Peripheral lung sections were co-immunostained with antibodies against the type II pneumocyte marker proSP-C (red) and type I pneumocyte marker Aqp5 (green). Nuclei were detected with DAPI. The immunostained area was quantified by counting the number of pixels present. Values represent means ± SE. Student's *t*-test; *P<0.05, **P<0.001. (**B–E**) TEM was performed on adult distal lungs from *Cby^+/+^* (**B, D**) and *Cby^−/−^* (**C, E**) mice. In the alveolar epithelium of *Cby^+/+^* mice, squamous type I pneumocytes and cuboidal type II pneumocytes containing lamellar bodies were observed. In *Cby^−/−^* lungs, the thickening of the cytoplasmic extension of type I cells was noted. Type II cells also exhibited morphological defects and frequently contained an increased number of lamellar bodies. Lipid-laden interstitial fibroblasts were often found in *Cby^−/−^* lungs (**C**). Black arrowheads point to microvilli (**D** and **E**). P_1_, type I pneumocytes; P_2_, type II pneumocytes; LB, lamellar bodies; C, capillaries; R, red blood cells; Av, alveolar airspace; M, mitochondria; asterisks, lipid droplets. Scale bars: (**A**) 50 µm; (**B, C**) 10 µm; (**D, E**) 5 µm.

The alveolar epithelium consists of two major cell types, squamous type I and cuboidal type II pneumocytes [29], [30]. Type I cells are directly involved in gas exchange, whereas type II cells secrete pulmonary surfactants and also serve as progenitors for type I cells. Immunostaining revealed that *Cby^−/−^* lungs had increased expression of pro-surfactant protein C (proSP-C), a marker for type II cells, in comparison with *Cby^+/+^* controls (Figure 6A). Quantitative assessment of proSP-C immunostaining by pixel counts showed a substantial difference between *Cby^+/+^* (1,437±477) and *Cby^−/−^* (47,660±4,796) mice (*P*<0.001). Conversely, expression of aquaporin 5 (Aqp5), a terminally differentiated type I cell marker, was reduced in *Cby^−/−^* lungs. Quantitative analysis by pixel counts also showed a significant difference between *Cby^+/+^* (34,320±8,715) and *Cby^−/−^* (4,454±2,066) mice (*P*<0.05).

To examine the ultrastructural morphology of the alveolar epithelial cells, transmission electron microscopy (TEM) was performed on lungs from adult *Cby^−/−^* and *Cby^+/+^* littermates. The normal alveolar epithelium is lined with cuboidal type II cells with characteristic lamellar bodies (secretory vesicles containing surfactants) and thin squamous type I cells that cover the majority of the alveolar surface area (Figure 6B and D). In *Cby^−/−^* mice, type II cells showed dramatic changes in morphology with a dark electron-dense appearance and some seemed to contain an increased number of lamellar bodies (Figure 6C and E). Additionally, type I cells exhibited a substantially thickened, disorganized morphology, suggestive of impaired differentiation. Interstitial fibroblasts often contained lipid droplets. In sum, these results demonstrate that loss of Cby leads to altered differentiation of alveolar epithelial cell lineages. At present, it remains unclear if this phenotype is directly associated with elevated Wnt/β-catenin signaling in *Cby^−/−^* mice.

### Low abundance of motile cilia in the proximal airways of *Cby^−/−^* mice

**Figure 7:**
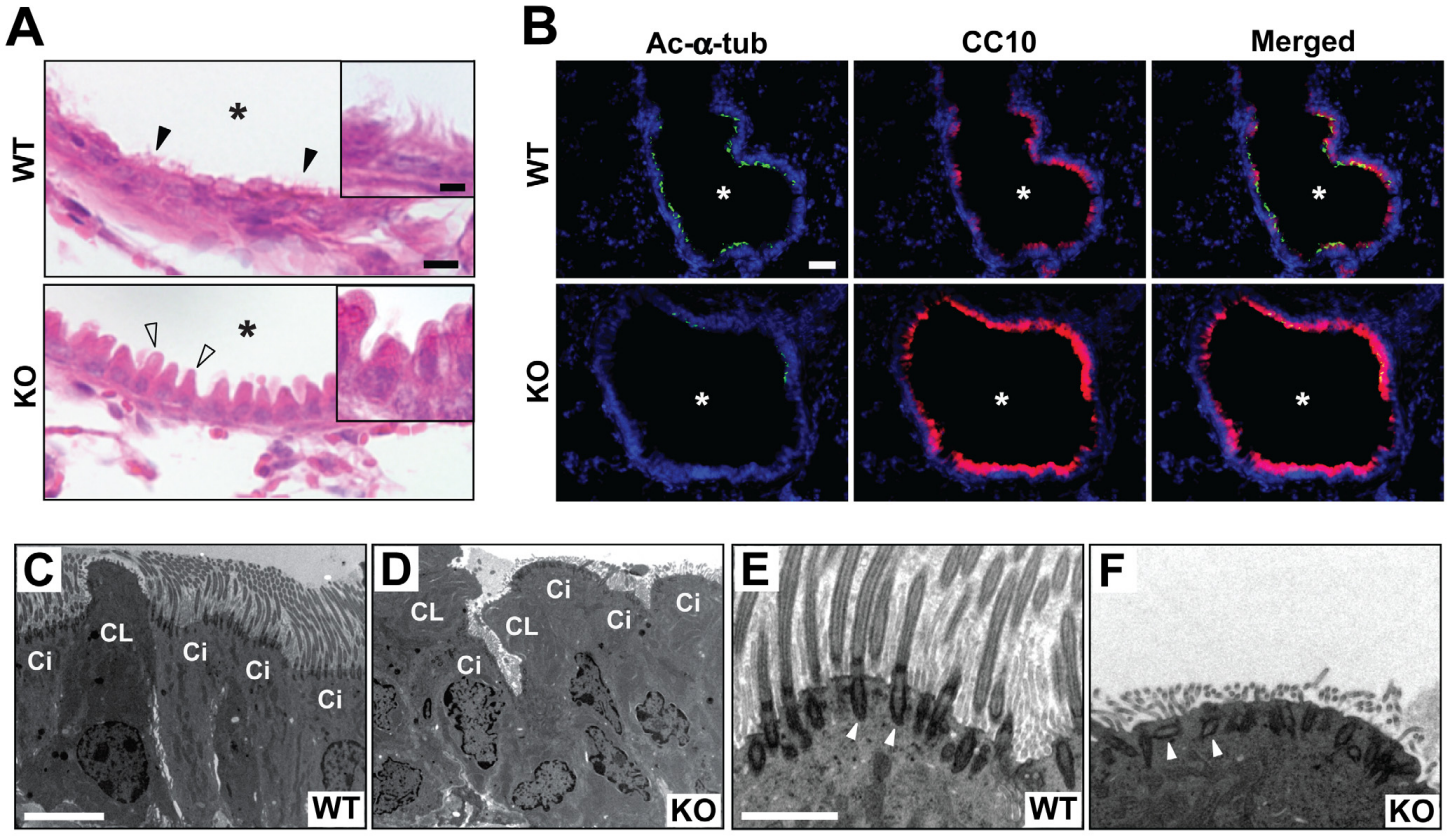
Proximal airway phenotypes of *Cby^−/−^* mice. (**A**) Lung airway sections from adult *Cby^+/+^* and *Cby^−/−^* mice were stained with H&E. Motile cilia were noticeable in the airway epithelium of *Cby^+/+^* mice (black arrowheads and inset) but not in that of *Cby^−/−^* mice. Atypical morphology of non-ciliated Clara cells was also observed in *Cby^−/−^* mice (white arrowheads and inset). Asterisks indicate the airway lumen. (**B**) Airway sections from adult *Cby^+/+^* and *Cby^−/−^* mice were double-labeled with antibodies against the ciliated cell marker acetylated α-tubulin (green) and Clara cell marker CC10 (red), and merged images are shown. Nuclei were stained with DAPI. (**C–F**) TEM was performed on adult proximal lungs from *Cby^+/+^* (**C, E**) and *Cby^−/−^* (**D, F**) mice. The airway epithelium of *Cby^+/+^* mice was lined with typical columnar ciliated and non-ciliated Clara cells. Strikingly abnormal morphology and disorganization of these cell types were seen in *Cby^−/−^* mice. In addition, ciliated cells had a marked paucity of motile cilia. High-magnification images of ciliated cells revealed that basal bodies (white arrowheads) were polarized perpendicular to the apical cell surface in *Cby^+/+^* mice (**E**), but frequently misoriented in *Cby^−/−^* mice (**F**). Ci, ciliated cells; CL, Clara cells. Scale bars: (**A**) 10 µm; (inset) 5 µm; (**B**) 50 µm; (**C, D**) 5 µm; (**E, F**) 500 nm.

In the epithelial lining of the proximal airways, there are two dominant cell types, secretory Clara cells and ciliated cells responsible for mucociliary clearance [29], [30]. The airway epithelium of adult *Cby^+/+^* mice showed a typical pseudostratified columnar morphology with cilia (black arrowheads and inset in Figure 7A). On the contrary, cilia were difficult to detect in *Cby^−/−^* mice. In addition, the apical protrusions of Clara cells were more prominent (white arrowheads and inset). The abnormal morphology of the airway epithelial cells was also confirmed by scanning EM (SEM) (Figure S3). Consistent with our histological and SEM data, in the large airway of adult *Cby^−/−^* mice, ciliary staining was greatly reduced as shown by immunofluorescent staining for acetylated α-tubulin (Figure 7B). In sharp contrast, there was a dramatic increase in staining for the Clara cell marker CC10 in *Cby^−/−^* lungs. It should be noted, however, that we found no evidence of intermediate cell types co-expressing CC10 and the ciliated cell marker Foxj1 in the airway epithelium of adult *Cby^−/−^* mice (data not shown).

At the ultrastructural level, a typical columnar epithelium composed of Clara cells and ciliated cells was observed in the proximal airway epithelium of control *Cby^+/+^* mice (Figure 7C). On the other hand, irregular cell shape and arrangement were readily noticeable in the *Cby^−/−^* airway epithelium (Figure 7D). We also found that ciliated cells were poorly differentiated with strikingly fewer ciliary projections although the axonemal ultrastructure of existing cilia was normal in *Cby^−/−^* mice (Figure S4). Interestingly, we noticed that some basal bodies, from which cilia extend, were positioned distantly from the apical plasma membrane and misoriented (compare white arrowheads in Figure 7E and F). These observations concur with our previous work showing that nasal ciliated cells in *Cby^−/−^* mice have a paucity of motile cilia with apparent basal body docking defects [23]. Our findings indicate that Cby is required for proper differentiation of airway epithelial cells.

### Cby is a direct target for Foxj1

**Figure 8:**
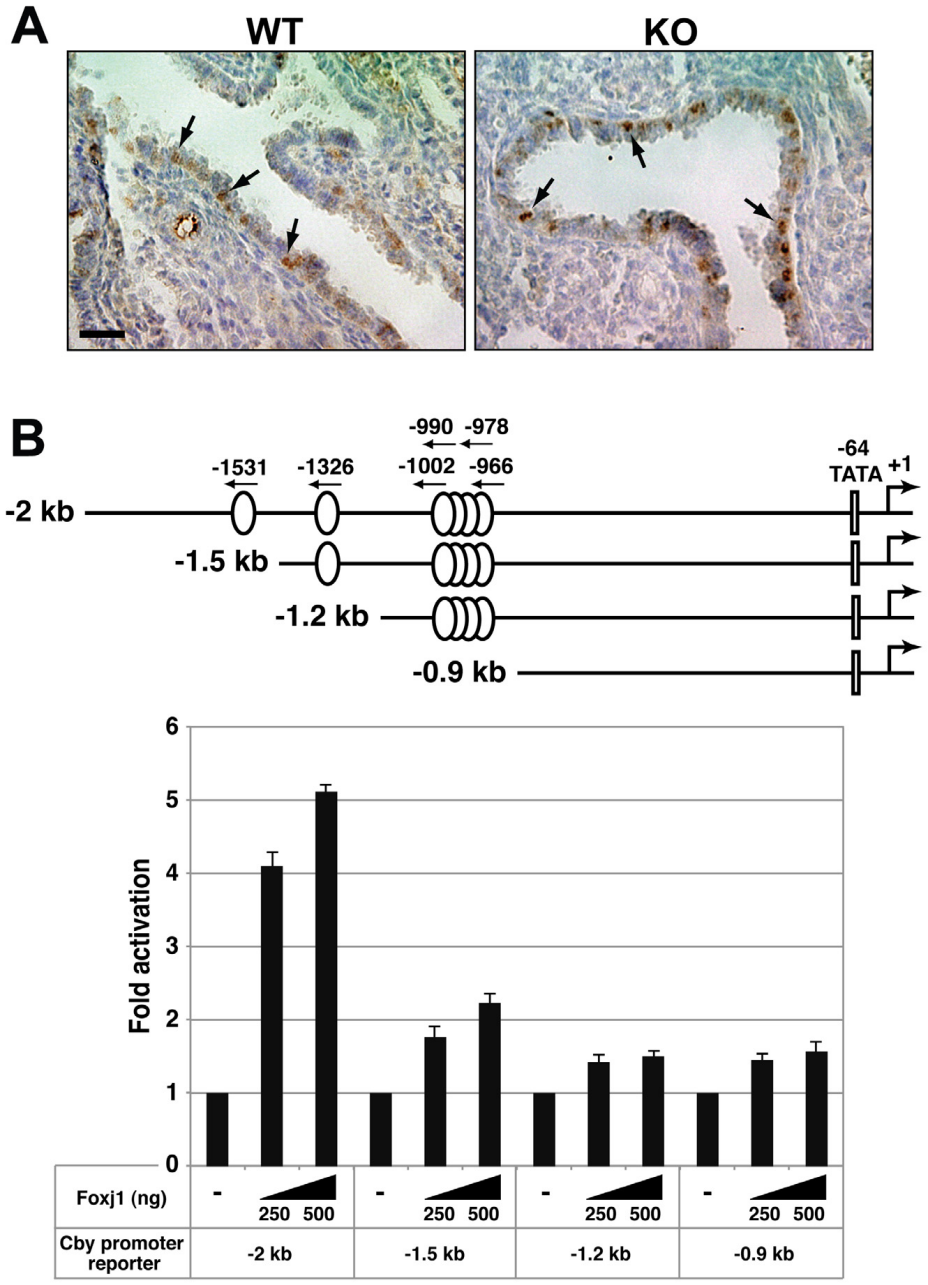
Cby is a direct target for Foxj1. (**A**) Foxj1 is expressed in *Cby^−/−^* lungs. Airway sections from E15.5 embryos were immunostained with anti-Foxj1 antibody, followed by hematoxylin counterstain. Arrows indicate positive nuclear staining of Foxj1. Scale bar, 50 µm. (**B**) Foxj1 directly activates Cby expression. Sequence analysis revealed 6 putative Foxj1-binding sites within the 2-kb mouse Cby promoter region (ovals). A predicted TATA box (TATGAA) was found at -64. The start of a mouse Cby cDNA sequence (GenBank accession number NM_028634) was tentatively designated as +1. The 5′ promoter deletion constructs are also illustrated. For luciferase reporter assays, HEK293T cells were transfected with 100 ng of each Cby promoter construct with the indicated amounts of a Foxj1 plasmid. Luciferase activity was measured 24 h after transfection and normalized to Renilla luciferase activity used as an internal control. The basal luciferase value of each Cby promoter reporter was set as 1. Transfections were done in triplicate, and the means ± SD are shown.

The ciliary phenotypes of *Cby^−/−^* mice and high expression of Cby in ciliated cells are reminiscent of those associated with the master ciliogenesis transcription factor Foxj1. Foxj1 is expressed in respiratory ciliated cells, and drives the motile ciliogenesis program by directly stimulating expression of various ciliogenesis genes including dyneins [31], [32], [33], [34]. This prompted us to investigate whether Foxj1 is expressed in *Cby^−/−^* mice. In mouse lungs, expression of Foxj1 begins at E15.5, before the appearance of cilia, in differentiating ciliated cells [33]. As shown in Figure 8A, Foxj1 was detectable in the airway epithelial cell nuclei of E15.5 *Cby^−/−^* lungs. These results imply that Cby lies downstream of Foxj1 in ciliated cells.

The above lines of circumstantial evidence raise the intriguing possibility that Foxj1 regulates Cby expression in airway ciliated cells. Inspection of the 2-kb mouse Cby promoter region revealed 6 putative Foxj1-binding sites, which closely match the proposed consensus sequence (Figure 8B) [35]. We also found three potential Foxj1-binding sites within the 2-kb 5′-flanking region of the human *Cby* gene (data not shown). To directly test if Foxj1 activates Cby expression, we performed luciferase reporter assays in HEK293T cells using a Cby promoter-luciferase construct harboring the 2-kb enhancer region of the mouse *Cby* gene [36]. Indeed, Foxj1 stimulated luciferase activity in a dose-dependent fashion (Figure 8B). Next, we generated a series of 5′ promoter deletion constructs to assess the importance of the putative Foxj1-binding sites. Strikingly, deletion of the most distal Foxj1-binding site (-1531) largely abrogated Foxj1-dependet activation. Further promoter deletions showed minimal changes in Foxj1 responsiveness, suggesting that the distal Foxj1-binding site is crucial for activation by Foxj1. These data indicate that Foxj1 positively regulates Cby expression during ciliated cell differentiation, thereby placing Cby in the motile ciliogenesis program.

### *Cby^−/−^* mice show abnormal respiratory mechanics

**Figure 9:**
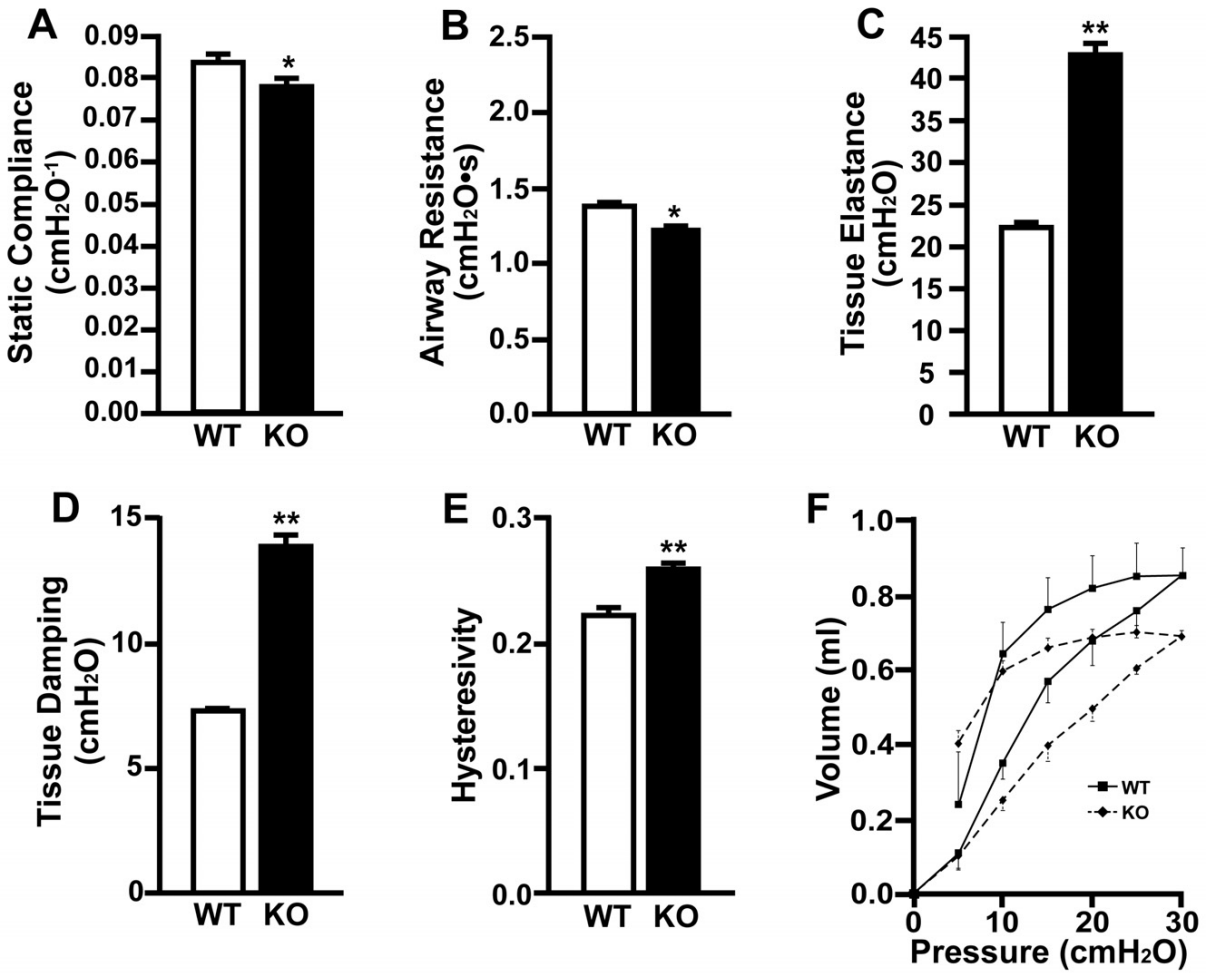
Abnormal pulmonary mechanics in adult *Cby^−/−^* animals. (**A–E**) Static compliance (**A**), airway resistance (**B**), tissue elastance (**C**), tissue damping (**D**) and hysteresivity (**E**) were measured with positive end-expiratory pressure (PEEP) at 0 cm H_2_O in 10- to 13-week-old *Cby^−/−^* (*n* = 5) and control *Cby^+/+^* (*n* = 5) mice. Similar results were obtained at a PEEP of 3 cm H_2_O (data not shown). Values were determined by fitting the constant-phase model to measurements of respiratory input impedance (*Zrs*) from each genotype. All measures were normalized by multiplication by total lung capacity (TLC) except for static compliance that was normalized by division by TLC. Values are means ± SE. Student's *t*-test; *P<0.05, **P<0.001. (**F**) Pressure-volume (*PV*) curve analysis was performed with a PEEP of 0 cm H_2_O on adult *Cby^−/−^* (*n* = 5) and *Cby^+/+^* (*n* = 5) littermates. Similar results were obtained at a PEEP of 3 cm H_2_O (data not shown). All measures were normalized by division by TLC. Values represent means ± SE. Student's*t*-test, *P*<0.05.

In order to investigate the potential effects of the observed structural defects in *Cby^−/−^* lungs on lung function, pulmonary function tests were performed. Static compliance (*Cst*), airway resistance (*Raw*), tissue elastance (*H*), tissue damping (*G*) and hysteresivity (*η*) were measured (Figure 9). The static compliance, which reflects elastic recoil at a given pressure, was reduced in *Cby^−/−^* mice compared to that in *Cby^+/+^* controls (*P*<0.05) (Figure 9A). Airway resistance, a frequency-independent Newtonian resistance, was also decreased in *Cby^−/−^* mice (*P*<0.05) (Figure 9B). On the other hand, tissue elastance, which reflects the energy conservation in lung tissues, was substantially increased in *Cby^−/−^* lungs (*P*<0.001) (Figure 9C). Tissue damping, which reflects the energy dissipation, was also increased in *Cby^−/−^* mice relative to that in *Cby^+/+^* mice (*P*<0.001) (Figure 9D). Lastly, hysteresivity, a reflection of inhomogeneities and structural changes in the lungs, was increased in *Cby^−/−^* mice (*P*<0.001) (Figure 9E). These pulmonary function data clearly indicate that targeted disruption of the *Cby* gene leads to perturbations in normal lung function.

The pressure-volume (*PV*) curves for each genotype are presented in Figure 9F. As expected, *Cby^+/+^* lungs exhibited normal *PV* relationships. In contrast, *Cby^−/−^* lungs did not distend as easily, demonstrating relatively small changes in volume with the same increments in applied transpulmonary pressure both initially and at high pressures. These results are consistent with the notion that *Cby^−/−^* lungs have increased stiffness and are less compliant.

## Discussion

Cby was originally identified as a conserved Wnt/β-catenin antagonist [19]. It directly binds to the C-terminal activation domain of β-catenin and inhibits β-catenin-mediated transcriptional activation [20], [21], [22]. More recently, we showed that Cby localizes to the base of motile cilia and controls ciliogenesis in the nasal epithelium of mice [23]. In this report, we demonstrate that genetic ablation of the *Cby* gene results in perturbed postnatal lung maturation with reduced proliferation and impaired differentiation of pulmonary epithelial cells, leading to alterations in the mechanical properties of the lungs. Thus, Cby plays an essential role in the proper development and function of the postnatal lung.

During embryonic and early postnatal lung development, Cby protein is ubiquitously expressed and intensely localizes to centrosome/basal body microtubules in undifferentiated lung epithelial cells (Figure 2). In adult lungs, Cby localization appears to be restricted specifically to the distal end of basal bodies in airway ciliated cells as detected by immunofluorescent staining. However, given the fact that various cell types are affected in *Cby^−/−^* adult lungs, we suspect that Cby protein may be present at low levels in other subcellular compartments of different cell types since Cby protein is able to shuttle between the nucleus and cytoplasm [22]. Alternatively, the transient localization of Cby at the centrosome/basal body in progenitor cells might be necessary for the normal differentiation of lung epithelial cell lineages.

The architectural abnormality of the lung parenchyma is one of the most prominent lung phenotypes resulting from the inactivation of Cby. Prior to the alveolarization stage (P5-P30; [2]), there are no obvious structural differences between *Cby^−/−^* and *Cby^+/+^* lungs (Figure 3, E18.5 and P4). However, there is a progressive reduction in the complexity of the parenchymal tissue of the developing *Cby^−/−^* lung during alveolarization. In the adult lung, the number of alveoli is significantly reduced in *Cby^−/−^* mice compared to *Cby^+/+^* controls (Figure 4). In line with these findings, we also observed an increase in the distance between alveolar walls, coincident with a decrease in the lung parenchymal tissue in *Cby^−/−^* mice. Our data suggest that the alveolar phenotype of *Cby^−/−^* mice results, at least in part, from reduced proliferation and altered differentiation of epithelial cells rather than septation defects (Figures 5 and 6). The precise molecular basis underlying the reduced complexity of the *Cby^−/−^* lung parenchyma remains elusive. In this regard, it is noteworthy that conditional activation of β-catenin specifically in the developing lung epithelium causes airspace enlargement with abnormal epithelial cell differentiation [15], implying that impaired alveolarization in *Cby^−/−^* mice is associated with up-regulation of β-catenin signaling. Indeed, we found that there is a chronic mild elevation of β-catenin signaling in *Cby^−/−^* lungs (Figure 1). This relatively mild effect of Cby deficiency on β-catenin signaling activity might be explained by the presence of two Cby homologues in mammals, Cby2 (also called Nurit [37]) and Cby3. Mouse Cby2 and Cby3 share 28% and 36% identity (47% and 57% similarity), respectively, with mouse Cby. Therefore, it is plausible that the Cby family members play redundant roles in regulating Wnt/β-catenin signaling.

The phenotypes of *Cby^−/−^* mice are reminiscent of some clinical features of primary ciliary dyskinesia (PCD). PCD is a rare genetically heterogeneous disorder characterized by dysfunctional motile cilia [24], [25]. Approximately 40% of PCD patients have mutations in the *DNAI1* and *DNAH5* genes that encode outer dynein arm components of ciliary axonemes. In addition, a small fraction of PCD patients were reported to carry mutations in 6 other genes, while the remaining causative genes are unidentified [25]. PCD patients manifest impaired mucociliary clearance and are therefore predisposed to recurrent infections including rhinitis, sinusitis, bronchitis and otitis media. Similarly, *Cby^−/−^* mice suffer from chronic upper respiratory infection and otitis media [23], but no clear signs of infection were observed in their lung. This is consistent with our previous findings that *Cby^−/−^* mice are able to clear bacteria from the lungs when challenged with *Pseudomonas aeruginosa* [23], suggesting the existence of cilia-independent defense mechanism (s) in murine airways. Another intriguing pulmonary pathology of *Cby^−/−^* mice is the significant increase in airway luminal area as revealed by morphometric analysis (Figure 4), presumably leading to a decrease in airway resistance (*Raw*) (Figure 9B). At present, it remains unknown if this is attributable to an increase in airway diameter, airway number or both. However, it is worth pointing out that bronchiectasis, bronchial widening, is a common complication associated with PCD although it is thought to be mainly caused secondarily by recurrent respiratory tract infection [24], [25].

*Cby^−/−^* mice show a marked paucity of motile cilia in the nasal epithelium [23] as well as in the airway epithelium (Figure 7). Consistent with this phenotype, Cby protein is highly enriched at the ciliary base, indicating that Cby plays a fundamental role in motile ciliogenesis. The exact mechanism of how Cby regulates ciliogenesis awaits further investigation. However, it is tempting to speculate that Cby protein at the distal end of basal bodies facilitates their migration and/or anchoring to the apical plasma membrane. Several components of the Wnt/β-catenin pathway including APC, Axin, Dishevelled and β-catenin have been reported to localize to centrosomes/basal bodies [38], [39], [40]. At present, whether Wnt/β-catenin signaling plays an active role in formation/function of cilia is poorly understood. Likewise, it remains to be seen if ciliary Cby is relevant to β-catenin signaling. Finally, we provide evidence that Cby expression is directly activated by Foxj1 (Figure 8). The phenotypic similarities of apparent basal body defects in ciliated cells of *Cby^−/−^* and *Foxj1^−/−^* mice suggest that Cby is a major downstream target for Foxj1.

In summary, we have shown that targeted disruption of the *Cby* gene encoding a Wnt/β-catenin antagonist affects postnatal lung maturation, resulting in abnormal lung function. The phenotypic features of *Cby^−/−^* mice may provide a valuable model system for studying PCD and potentially other cilia-related disorders.

## Materials and Methods

### Mouse strains

The creation and genotyping of *Cby* knockout mice and BAT-gal reporter mice have been described previously [23], [26]. *Cby^+/−^* mice were crossed with BAT-gal mice to obtain *Cby^+/−^* progeny carrying the BAT-gal transgene. These animals were then crossed with *Cby^+/−^* mice to produce BAT-gal transgenic *Cby^+/+^* and *Cby^−/−^* embryos. For timed matings, noon of the day when a vaginal plug was observed was considered E0.5. Animals were housed in pathogen free conditions, and all experimental procedures involving mice were approved by the Institutional Animal Care and Use Committee of the SUNY at Stony Brook (Protocol 1393).

### RNA extraction, RT-PCR and real-time PCR

Total RNA was purified from murine lungs using the RNeasy Mini Kit (Qiagen) with DNase treatment. For RT-PCR in Figure S1A, cDNA synthesis was performed with oligo(dT) primers using the ThermoScript RT-PCR System (Invitrogen) according to the manufacturer's instructions. The primer sequences were as follows: Cby forward, 5′-CGTTTCCTCACTGAGTTAGG-3′; Cby reverse, 5′-TAGTCTGCTAATCTGACGGG-3′; GAPDH-1 forward, 5′-ACCACAGTCCATGCCATCAC-3′; GAPDH-1 reverse, 5′-TCCACCACCCTGTTGCTGTA-3′. Quantitative real-time PCR analysis was performed using the iScript One-Step RT-PCR Kit with SYBR Green (BioRad) on the MiniOpticon Real-Time PCR Detection System (BioRad). The following primer pairs were used: cyclin D1 forward, 5′-TGTTCGTGGCCTCTAAGATGAAG-3′; cyclin D1 reverse, 5′-AGGTTCCACTTGAGCTTGTTCAC-3′; axin2 forward, 5′-CTCCCCACCTTGAATGAAGA-3′; axin2 reverse, 5′-ACATAGCCGGAACCTACGTG-3′; GAPDH-2 forward, 5′-TCAACAGCAACTCCCACTCTTCCA-3′; GAPDH-2 reverse, 5′-ACCCTATTACTGTAGCCGTATTCA-3′. The level of transcripts for GAPDH was used as an internal standard. The amplification steps consisted of 10 min at 50°C and 5 min at 95°C, followed by 40 cycles of denaturation for 10 sec at 95°C and annealing/extension for 30 sec at 58°C. All samples were analyzed in triplicate, and the relative gene expression was calculated according to the comparative threshold cycle (ΔΔC_t_) method [41].

### Western blotting

For detection of Cby protein in Figure S1B, lung tissue lysates were prepared using TRIzol reagent (Invitrogen). Equal amounts of protein samples were loaded onto a 15% SDS-PAGE, and subjected to immunoblotting using rabbit anti-Cby antibody [19].

### Immunohistochemistry

Lung samples were embedded with the Cryo-Gel medium (Instrumedics), and frozen sections processed for immunostaining as described previously [23]. The following primary antibodies were used: polyclonal Cby (1∶500; [23]), monoclonal Cby 8-2 (1∶100; Santa Cruz Biotechnology), acetylated α-tubulin (1∶500; Sigma-Aldrich), CC10 (1∶500; gift from Dr. Barry Stripp), proSP-C (1∶500; gift from Drs. Avinash Chander and Susan Reynolds), aquaporin 5 (1∶500; Sigma-Aldrich) and p180 (1∶700; Covance). Antigen-antibody complexes were detected with Alexa Fluor 488- or 568-conjugated secondary antibodies (1∶500; Invitrogen). The sections were then stained with DAPI (Sigma-Aldrich) and mounted using Fluoromount-G (Southern Biotechnology Associates). Foxj1 was detected on paraffin sections with anti-Foxj1 antibody (1∶100; [42]) using the mouse-on-mouse (MOM) kit (Vector Laboratories), followed by hematoxylin counterstain. Images of representative fields were acquired using an Olympus BX61 microscope equipped with a Cooke Sensicam QE CCD camera. The pixel intensity of proSP-C or aquaporin 5 immunofluorescence (Figure 6A) was quantified in 5 random fields from each of 3 independent sections using the SlideBook imaging software (Intelligent Imaging Innovations).

### Histological analysis

Mice were euthanized by CO_2_ asphyxiation, and the trachea was exposed and cannulated. Lungs were then inflation-fixed at a pressure of 20 cm H_2_O for 24 h in 4% methanol-free formaldehyde (Polysciences) in PBS, pH 7.4. Right lung lobes were removed, dehydrated through a series of increasing ethanol washes and embedded in paraffin. Five-µm sections were generated, dehydrated and stained with hematoxylin and eosin (H&E) as well as Masson's trichrome (Sigma-Aldrich) to observe lung architecture and the presence as well as distribution of collagen, respectively.

### BrdU incorporation assay

Cell proliferation was quantified by BrdU labeling. Briefly, BrdU (10 mg/ml) was injected intraperitoneally into P11 mice at a dose of 5 µl/g body weight. After 2 h, lungs were collected and embedded with the Cryo-Gel medium (Instrumedics). Frozen sections were post-fixed with methanol/acetone (1∶1), and processed for immunofluorescent staining with anti-BrdU antibody (1∶250; Accurate Chemicals), followed by Alexa Fluor 568-conjugated secondary antibody (1∶500; Invitrogen). The number of BrdU-positive cells was counted in 5 random 60× objective fields (n = 3 per genotype) and the average number of labeled cells per field was calculated.

### Lung morphometry

Morphometric measurements were performed on inflation-fixed *Cby^+/+^* and *Cby^−/−^* lungs as described previously [43]. At least five representative sections from the right lobes of each sample were randomly chosen at 250-µm intervals and stained with H&E. Twenty images from non-overlapping parenchymal fields in each lung section were captured at a 40× objective magnification. A square lattice grid of 121-test points was then superimposed on each image to evaluate saccular airspace and wall, parenchyma, and airway lumen by point-counting morphometry by two investigators. Parenchyma was defined as the gas-exchanging compartment that contained the airspaces (saccule ducts and saccules). Airways consisted of conducting airways to the level of the terminal bronchioles. Alveolar complexity of the lung was assessed by counting the number of individual saccules in each field, and measuring the mean linear intercept (Lm) (distance between alveolar walls).

### Respiratory function tests and pressure-volume (*PV*) curve analysis

Pulmonary mechanics were assessed on adult mice using a computer-controlled ventilator flexiVent (SCIREQ, Montreal, PQ, Canada) as described previously [44], [45]. The mice were anesthetized with intra-peritoneal pentobarbital (90 mg/kg) and the trachea was dissected free of surrounding tissue and cannulated with a 20-gauge catheter. The animals were then connected to flexiVent and ventilated with a tidal volume of 10 ml/kg; inspiratory:expiratory ratio of 66.67%, respiratory rate of 150 breaths/min, and maximum pressure of 30 cm H_2_O, with a positive end-expiratory pressure (PEEP) of 0 and 3 cm H_2_O. PEEP was controlled by submerging the expiratory limb from the ventilator into a water trap. This ventilator permits us to measure lung function by using a modification of the low-frequency forced oscillation technique [46]. Respiratory input impedance (*Zrs*) was measured and interpreted in terms of the constant-phase model [47] to obtain airway resistance (*Raw*) or tissue damping (*G*) and elastance (*H*). Hysteresivity measured to describe the mechanical coupling between tissue damping and elastance was calculated as *G*/*H*.

Analysis of *PV* curves was conducted as follows. Starting at a functional residual lung capacity (FRC) defined by the PEEP, the flexiVent was programmed to apply inspiratory volume in stepwise fashion until a peak pressure of 30 cm H_2_O was reached, followed by stepwise expiration. At each step, ventilation was paused for 1 sec and plateau pressure (*P*) was recorded and related to the total volume (*V*) delivered to produce a quasi-static *PV* curve. Static compliance (*Cst*) was calculated from the slope of each curve as described previously [48]. *Zrs* and *PV* curve data were obtained in triplicate for statistical evaluation.

### Transmission electron microscopy (TEM) and scanning EM (SEM)

TEM was performed in the Central Microscopy Imaging Center, SUNY at Stony Brook. Briefly, mice were anesthetized intraperitoneally with 90 mg/kg ketamine and 10 mg/kg xylazine, and perfused transcardially with 2% paraformaldehyde and 2% EM-grade glutaraldehyde in PBS, pH 7.4. Lung tissue was dissected and post-fixed in 2% osmium tetroxide, dehydrated and embedded in Durcupan resin. Ultrathin sections of 80 nm were cut with a Reichert-Jung Ultracut E ultramicrotome and placed on formvar-coated slot copper grids. Sections were then counterstained with uranyl acetate and lead citrate, and analyzed by a FEI Tecnai12 BioTwinG^2^ electron microscope. Digital images were acquired with an AMT XR-60 CCD Digital Camera System and compiled using Adobe Photoshop software. SEM was performed essentially as described [23].

### BAT-gal and luciferase reporter assays

Lung extracts were prepared from E15.5 and E16.5 embryos using the Galacto-Light Plus System (Applied Biosystems) according to the manufacturer's instructions, and β-galactosidase activity was measured using a Monolight 2010 luminometer (BD Biosciences).

The -2-kb mouse Cby promoter luciferase reporter in the pGL3-Basic backbone has been previously published [36]. This -2-kb reporter was double-digested by MluI and BglII, AgeI or BstBI, blunt-ended and self-ligated to generate the -1.5-kb, -1.2-kb or -0.9-kb deletion constructs. For luciferase reporter assays, HEK293T cells were seeded onto 12-well tissue culture dishes, cultured overnight and then transfected with appropriate combinations of plasmids in triplicate using Expressfect (Denville Scientific). Luciferase activities were measured using the Dual-Luciferase Reporter Assay System (Promega) and a Berthold luminometer as described previously [21], [22]. A Renilla luciferase vector (pRL-TK) was cotrasfected to normalize transfection frequency.

### Statistical analysis

Unpaired Student's *t*-test was performed to determine statistically significant differences among different genotype groups. Values for all measurements were expressed as means ± SE, and *P* values of <0.05 were considered significant.

## Supporting Information

Figure S1 Cby is expressed throughout lung development. (A) The temporal expression of Cby mRNA was analyzed by RT-PCR in lung tissue samples from embryonic day (E) 14.5, E17.5, postnatal day (P) 7, P21 and adult Cby+/+ mice, and adult Cby-/- mice (negative control). GAPDH was used as a loading control. (B) Cby protein was detected in the adult lung. Equal amounts of lung homogenates (50 μg) from Cby+/+ and Cby-/- adult animals were loaded onto a 15% SDS-PAGE, and subjected to western blotting using anti-Cby antibody.
(0.76 MB TIF)

Figure S2 Cby protein is present in multiple cell types in embryonic lungs. (A) Peripheral lung sections from E17.5 Cby+/+ lungs were co-immunostained for Cby (red) and the alveolar type II cell marker p180 (green). Arrows point to discrete dots of Cby staining seen in immature type II cells. (B) Lung airway sections from E16.5 Cby+/+ lungs were double-labeled with antibodies against Cby (red) and the ciliated cell marker Foxj1 (green). Arrowheads point to ciliated cell precursors with apical Cby signals. Nuclei were visualized by DAPI. The asterisk indicates the airway lumen. Note that Cby is observed in cell types other than type II and ciliated cell progenitors. Scale bar, 1 μm.
(6.94 MB TIF)

Figure S3 Scanning electron microscopy (SEM) of the lung airway epithelium. SEM images of adult proximal airways reveal a marked paucity of cilia and prominent apical protrusions of Clara cells in Cby-/- mice. Some contaminating red blood cells are present. Scale bar, 10 μm.
(7.55 MB TIF)

Figure S4 Axonemal ultrastructure of lung airway cilia appears normal in Cby-/- mice. Cross sections of bronchial cilia in adult Cby+/+ and Cby-/- mice were analyzed by transmission electron microscopy (TEM). Motile cilia in Cby-/- mice have an apparently normal axonemal ultrastructure with a typical 9+2 microtubular arrangement and dynein arms. Scale bar, 100 nm.
(7.76 MB TIF)
