# Supplementary figures and images for: Altered Lung Morphogenesis, Epithelial Cell Differentiation and Mechanics in Mice Deficient in the Wnt/β-Catenin Antagonist Chibby
Source: PLoS One. 2010 Oct 25;5(10):e13600. doi: 10.1371/journal.pone.0013600 (PMC2963606; doi:10.1371/journal.pone.0013600)

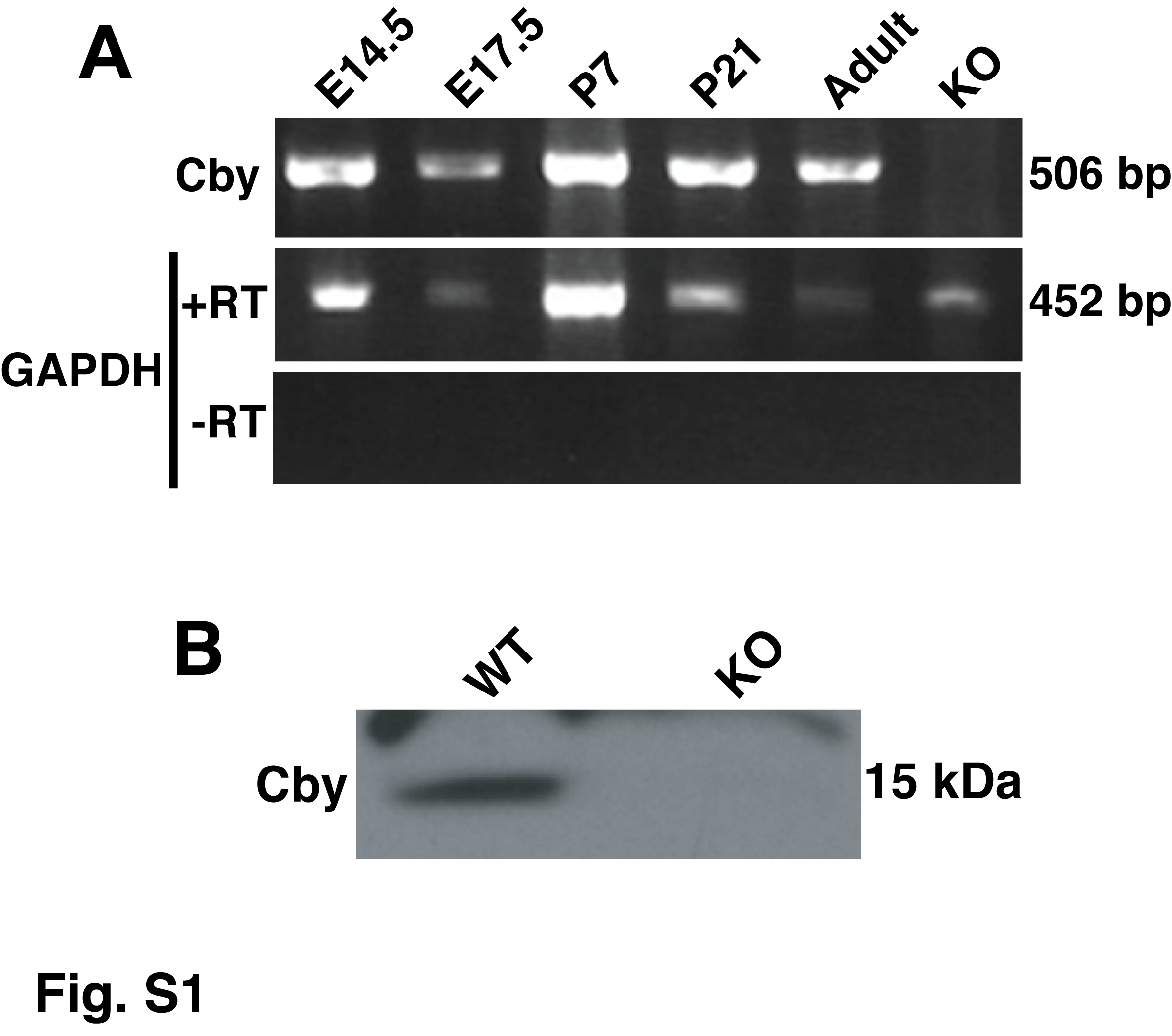

Supplement: Figure S1 — Cby is expressed throughout lung development. (A) The temporal expression of Cby mRNA was analyzed by RT-PCR in lung tissue samples from embryonic day (E) 14.5, E17.5, postnatal day (P) 7, P21 and adult Cby+/+ mice, and adult Cby-/- mice (negative control). GAPDH was used as a loading control. (B) Cby protein was detected in the adult lung. Equal amounts of lung homogenates (50 μg) from Cby+/+ and Cby-/- adult animals were loaded onto a 15% SDS-PAGE, and subjected to western blotting using anti-Cby antibody. (0.76 MB TIF) [file pone.0013600.s001.tif]

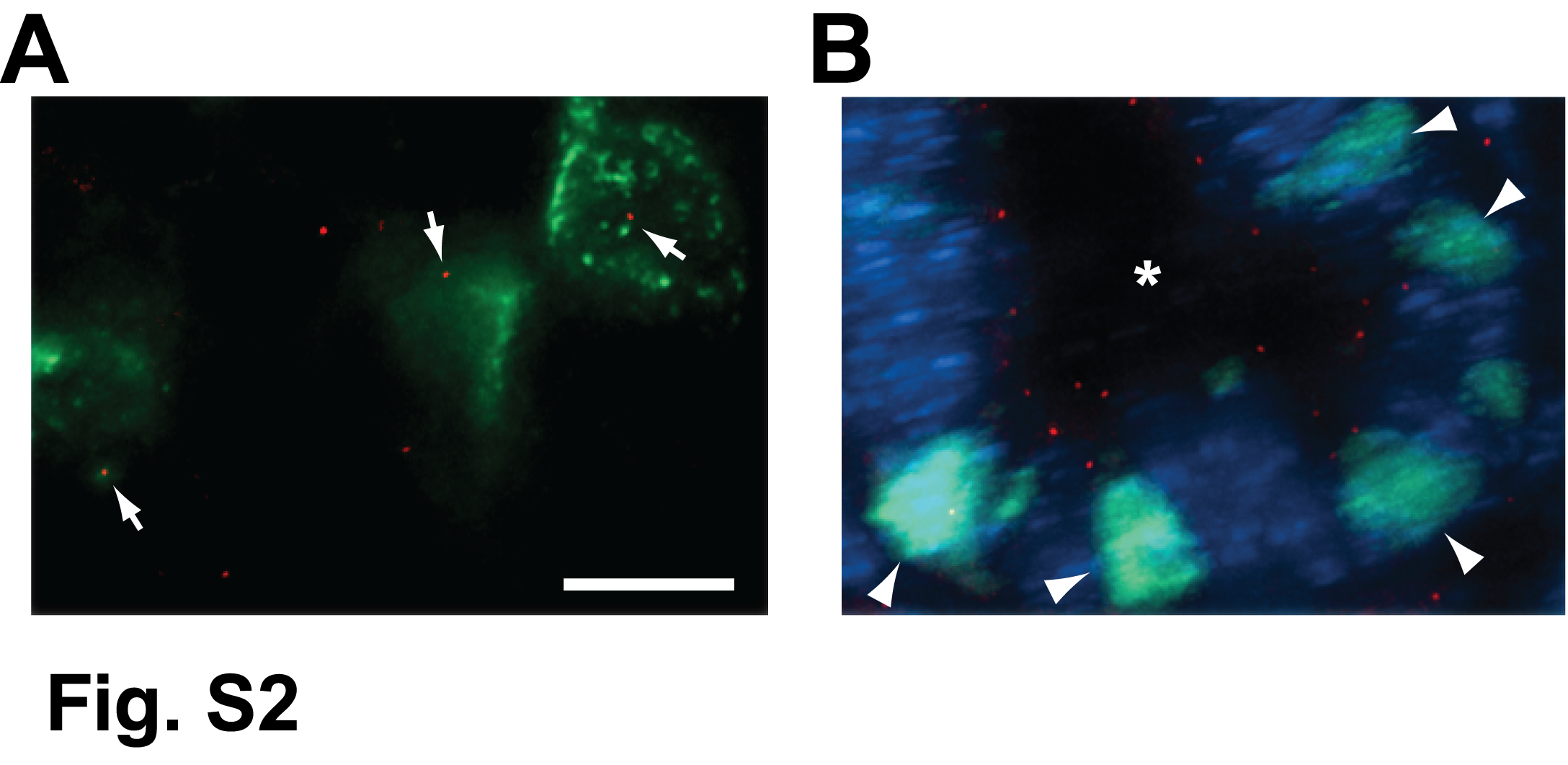

Supplement: Figure S2 — Cby protein is present in multiple cell types in embryonic lungs. (A) Peripheral lung sections from E17.5 Cby+/+ lungs were co-immunostained for Cby (red) and the alveolar type II cell marker p180 (green). Arrows point to discrete dots of Cby staining seen in immature type II cells. (B) Lung airway sections from E16.5 Cby+/+ lungs were double-labeled with antibodies against Cby (red) and the ciliated cell marker Foxj1 (green). Arrowheads point to ciliated cell precursors with apical Cby signals. Nuclei were visualized by DAPI. The asterisk indicates the airway lumen. Note that Cby is observed in cell types other than type II and ciliated cell progenitors. Scale bar, 1 μm. (6.94 MB TIF) [file pone.0013600.s002.tif]

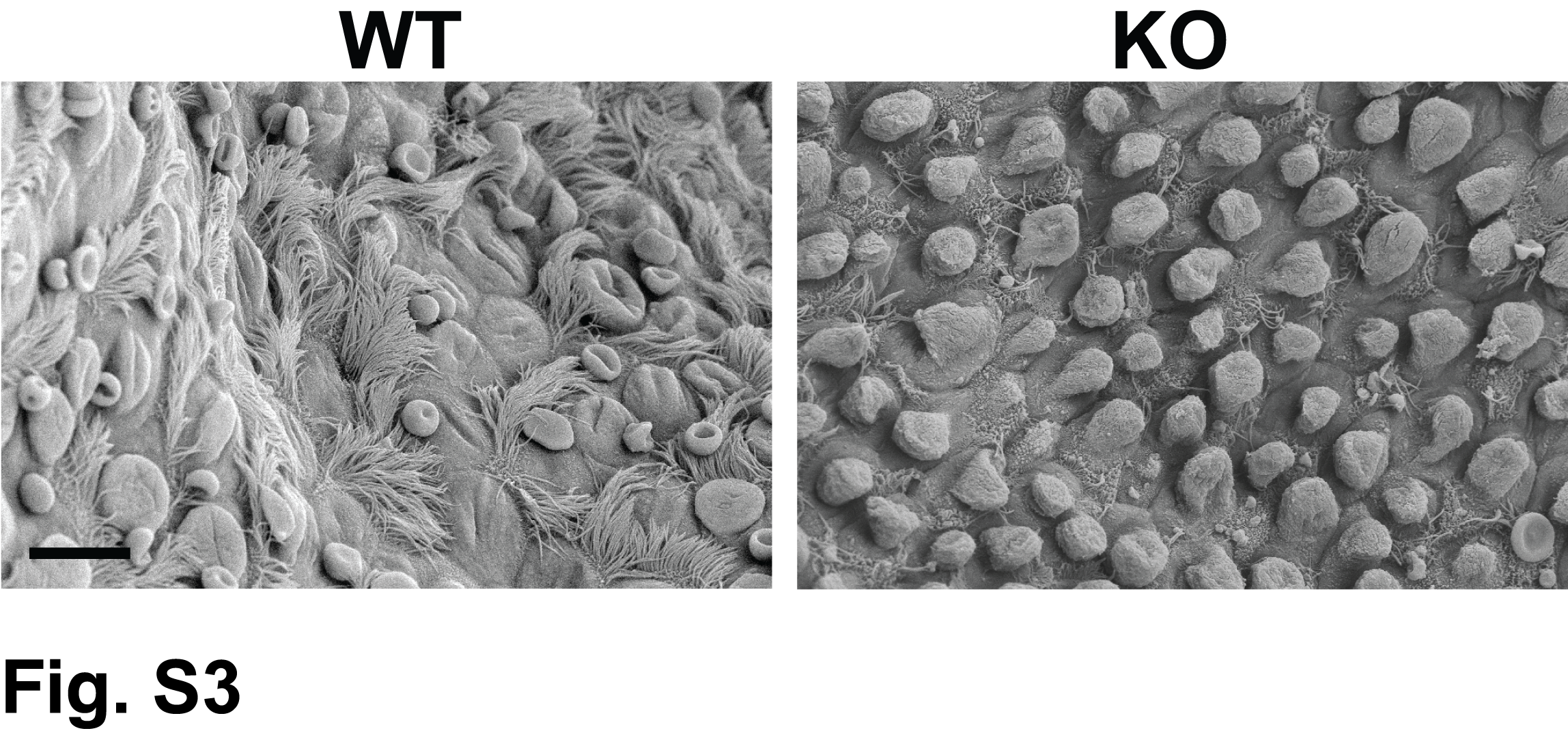

Supplement: Figure S3 — Scanning electron microscopy (SEM) of the lung airway epithelium. SEM images of adult proximal airways reveal a marked paucity of cilia and prominent apical protrusions of Clara cells in Cby-/- mice. Some contaminating red blood cells are present. Scale bar, 10 μm. (7.55 MB TIF) [file pone.0013600.s003.tif]

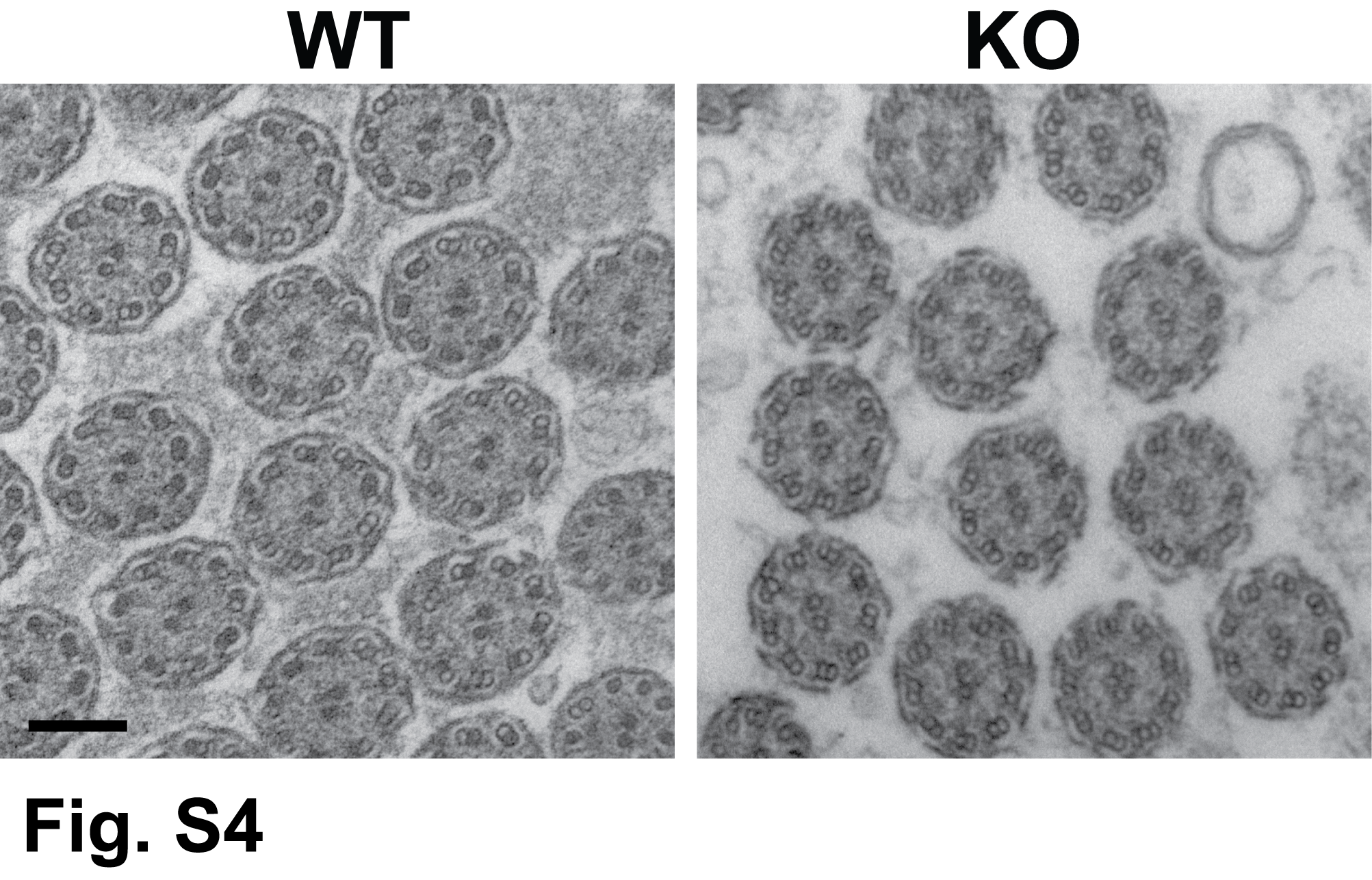

Supplement: Figure S4 — Axonemal ultrastructure of lung airway cilia appears normal in Cby-/- mice. Cross sections of bronchial cilia in adult Cby+/+ and Cby-/- mice were analyzed by transmission electron microscopy (TEM). Motile cilia in Cby-/- mice have an apparently normal axonemal ultrastructure with a typical 9+2 microtubular arrangement and dynein arms. Scale bar, 100 nm. (7.76 MB TIF) [file pone.0013600.s004.tif]
